# Work Environment, Stress, and Driving Anger: A Structural Equation Model for Predicting Traffic Sanctions of Public Transport Drivers

**DOI:** 10.3390/ijerph15030497

**Published:** 2018-03-12

**Authors:** Luis Montoro, Sergio Useche, Francisco Alonso, Boris Cendales

**Affiliations:** 1 FACTHUM Lab (Human Factor and Road Safety) Research Group, INTRAS (Research Institute on Traffic and Road Safety), University of Valencia, 46022 Valencia, Spain; luis.montoro@uv.es; 2 DATS (Development and Advising in Traffic Safety) Research Group, Faculty of Psychology, INTRAS (Research Institute on Traffic and Road Safety), University of Valencia, 46022 Valencia, Spain; francisco.alonso@uv.es; 3 Faculty of Economic and Administrative Sciences, El Bosque University, Bogotá 110121, Colombia; boriscendales@gmail.com

**Keywords:** working conditions, stress, job strain, driving stress, driving anger, risky road behavior, road misbehaviors, traffic sanctions

## Abstract

Public transport is an effective and sustainable alternative to private vehicle usage, also helping to reduce the environmental impact of driving. However, the work environment of public transport operators is full of adverse conditions, which, together with their high mileage, may increase the occurrence of negative safety outcomes such as traffic accidents, often preceded by risky road behaviors enhanced by stress, anger, and difficult operating conditions. The aims of this study were, first, to determine the association between work-related psychosocial factors and individual characteristics of public transport drivers and the rate of traffic sanctions they are subject to; and second, to assess the mediation of driving anger in this relationship. A sample of professional drivers (57.4% city bus, 17.6% taxi, and 25% inter-urban bus male operators) was used for this cross-sectional study, responding to a five-section survey including demographic data and driving-related factors, psychosocial work factors including job stress, driving stress, risk predisposition, and driving anger. The results of this study showed significant associations between work-related factors: measures of stress and self-reported rates of traffic fines. Second, it was found that driving anger mediates the associations between driving stress, risk predisposition, and traffic sanctions; and partially mediates the association between driving experience, hourly intensity, and job stress. This study supports the idea that traffic penalties reported by public transport rates are preceded by work-related, personality, and other individual factors that, when combined with driving anger, enhance the occurrence of road misbehavior that may affect overall road safety.

## 1. Introduction

Worldwide, the public transportation industry provides an essential service for the population, while it employs millions of people, most of them working as vehicle drivers.

It has been indicated as an effective way to reduce traffic density, the use of private vehicles and, by extension, of road accidents [1,2]. However, the benefits of the industry’s exponential growth, along with excessive global motorization, have come with a substantial price for its workers [3].

Vehicle operators in the transport sector industry may belong to the occupational group with the highest prevalence of job stress rates [4,5], and many environmental variables associated with the job of professional drivers, especially those working in the field of public transportation, have been addressed by different studies as typically adverse [6,7]. Several factors such as continuous time pressure, excessive physical demands, environmental overstimulation, problematic interactions with other road users, lack of social support at work, and irregular shifts [8,9] have been characterized as potential stress-related factors, which at the same time enhance the potential occurrence of negative outcomes in terms of health, safety, and performance [10,11,12]. Traffic accidents involving public transport vehicles constitute an undetermined but high proportion of the total road crashes involving fatalities and severe injuries, making such accidents an even more serious public health issue [3]. In this sense, it is worth emphasizing traffic safety when considering the occupational safety of public transport drivers. In other words, the improvement of factors influencing the risky driving of this occupational group may have positive effects on overall road safety.

### 1.1. Work Environment and Stress in Professional Drivers

As noted at the beginning of this manuscript, the typically adverse environmental and task conditions in which the work of public transport drivers takes place is characterized by harmful conditions that may affect their health and well-being [6,8,13]. First is their constant exposure to environmental overstimulation (e.g., noise, smog, variable light conditions) [5] and difficult ergonomic conditions [10]. Second, public transport drivers’ rest periods tend to be insufficient or just inadequate, bearing in mind dynamics such as shift work—commonly observed in the transport industry—that impede the scheduling of fixed recovery time to alleviate work-related fatigue (breaks are normally irregular) [14,15]. Thirdly, some studies have shed light on how drowsiness and fatigue constitute key risk factors related to professional drivers’ risky behaviors and traffic accidents [12,16]. In fact, the relationship between shift work/fatigue and negative safety outcomes is clearer in the field of transportation than s in any other industry [15,17].

The sum of all these difficult conditions, combined with a reduced capacity to make decisions and to keep a certain level of control over their work, may lead to job stress, which is one of the factors most frequently associated with risky road behavior among professional drivers, and may even predict a significant proportion of the potential occupational accidents and injuries transport workers are subject to, along with their associated elevated costs for transport companies [8,9]. Regarding the work stress approach used in this research, the Job Demand⎯Control (JDC) model [18] supports the assumption that work stress occurs when high psychological demands are combined with low rates of perceived control. In short, the job work stress indicator of the JDC model is commonly known as job strain. In this regard, several studies have associated job strain with different negative outcomes for transport workers, especially in the form of health complaints, chronic fatigue, and overall impaired job performance [19,20,21].

In the specific case of professional drivers, work stress is closely related to driving stress. If the job involves the operation of a vehicle, there is a likelihood that job demands may influence the drivers’ general attitudes, driving-related reactions, and observed behaviors on the road [9,12]. Thus, many of the factors related to the work environment and task design constitute common prevalent stressors [19,22]. A previous study found that around half of commercial drivers commonly experience adverse affective reactions during their work (driving) schedules [23]. However, due to the commonly observed lack of road training and awareness, driving stress is hardly recognized by most individuals.

Other empirical studies have found stress-related factors to be effective predictors of dangerous driving behaviors [24], which may impair driving performance in terms of producing more errors and traffic violations, predisposing drivers to an increased risk of suffering traffic accidents, and causing injury to other road users [12,23].

Beyond work and driving-related stress, a number of different factors may influence risk on the road. One of these factors is personality [24,25]. The role of personality in driving has a proven connection with driving style and may predispose drivers to higher risk in driving [26,27]. Also, individual differences in variables such as age, driving experience, and personality traits are important factors that may interact with stress, affecting the safety outcomes of drivers [9,24], including professional drivers of different types of vehicles. This is the case for driving anger [28].

### 1.2. Driving Anger and Risky Driving

Anger and aggressive expressions on the road constitute a very complex problem, considering that both are influenced by a large number of variables, including, of course, stress. In fact, apart from psychological traits, the clear majority of these factors are related to different social (relational) and cultural issues. This provides the possibility of studying and modifying such factors through empirical interventions aimed at strengthening their avoidance and, subsequently, considerably reducing the probability of road accidents related to the occurrence of aggressive behaviors [29,30]. A number of empirical studies have revealed that angry drivers present highly negative interactions with other road users, and are more likely to show aggressive expressions and risky behaviors on the road than less angry drivers [31,32].

A significant number of these studies have found that more accidents occur when drivers experience considerable rates of anger, leading them to express instrumental or hostile behaviors towards others, and to committing core errors and traffic violations [27,33,34,35]. Ergo, aggressive behaviors are positively associated with the probability of causing traffic accidents, making such behavior relevant to road safety and, of course, to the study of road crashes explained by human factors [36,37]. In this sense, while anger and aggressive driving can help explain a large proportion of global accidents, strategies such as road safety education and training, psychotherapy, and anger-management interventions may contribute to reducing crash rates and the risky behaviors preceding them [38,39].

### 1.3. The Current Study

Conceptually, this study is based on the work stress/emotion/counterproductive work behavior (S/E/CWB) model [40,41], according to which, counterproductive work behaviors (CWB), such as traffic infractions, are indirect responses to job stressors. From this perspective, environmental threats that jeopardize individual well-being (i.e., stressors) induce negative emotional responses such as anger and anxiety [40,42]. These emotions, which represent the primary response to stressful situations, in turn, motivate and energize behavioral responses (e.g., fight or flight). Several studies [41,43] have found evidence supporting the mediating role of emotions in the association between organizational constraints and CWBs (e.g., organizational aggression, cynicism, low task involvement, bullying/mobbing).

Bearing in mind that in the S/E/CWB model, emotions are a mechanism that links environmental stressors with behavior, this study hypothesizes that driving anger mediates the association between the stressors of the work environment (job strain, driving stress and driving intensity) and the sanctioned driving misbehaviors (traffic fines) of professional drivers. The model also suggests that there are individual factors that may interfere with the flow from environment appraisal, to negative emotion, to CWB [43]. Therefore, it is also expected that the driving experience and risk predisposition (based on personality) of professional drivers will have a direct effect on traffic sanction rates. In this regard, most similar studies have used accident or crash rates as a dependent variable but, in this case, we addressed the problem of traffic sanction rates of professional drivers as a potential result with theoretical plausibility. Thus, bearing in mind the methods employed and the scope, the main strength of this study is the predictive modeling of traffic sanctions, that is, risky behaviors that do not necessarily lead to a traffic accident, but do threaten occupational and road safety.

Specifically, the aims of this study were, first, to examine the association between work-related psychosocial stressors, individual characteristics of public transport drivers, and their sanctioned driving misbehaviors (i.e., traffic sanctions) in the last two years; and second, to assess the mediation of driving anger in this relationship.

According to the aforementioned objectives, the hypotheses of this study were: First, that work-related stressors, some individual characteristics of drivers, and their traffic sanction rates will be significantly associated, considering the described relationships between working conditions, stress, and risky driving that have arisen in other studies [12,23,24]. Second, that driving anger will link work stressors, hourly intensity, individual factors, and traffic sanctions, bearing in mind that driving anger has been related to stressful conditions of driving as a factor that enhances its experience and expression [28], and to greater odds of negative traffic outcomes as a result [33]. 

## 2. Materials and Methods

### 2.1. Sample

This study involved a sample of professional drivers working in Colombian public transport companies: 448 (57.4%) of them were city bus drivers, 195 (17.6%) were taxi drivers, and the remaining 137 (25%) were inter-urban bus operators. Bearing in mind their underrepresentation in this occupational group (approximately 97% of the preliminary full sample was male), and to avoid any potential gender-related bias, women (*n* = 25, from an initial sample of *n* = 805) were excluded from the final sample, resulting in an actual size of *n* = 780 male public transport drivers.

### 2.2. Instrument

The questionnaire was written in Spanish and consisted of five sections: the initial section addressed individual/demographic variables, such as age, gender, and city of residence, and driving-related factors, such as driving experience (measured in years), driving frequency, and length of time (i.e., hours per week; weekday and weekend driving) to calculate hourly intensity and the number of traffic sanctions or fines received in a period of (the last) two years.

The second section assessed exposure to work stress, using the Colombian version of Karasek’s Job Content Questionnaire (JCQ) [18], made up of 27 items grouped into six scales: supervisor support (four items, α = 0.87), peer support (four items, α = 0.79), skill discretion (six items, α = 0.75), decision authority (three items, α = 0.69), psychological demands (six items, α = 0.66), and job insecurity (four items, α = 0.53). Decision latitude or control was calculated as the sum of use of skills and decision-making, whereas job strain was calculated as the ratio between psychological demands and decision latitude (demands/control). For this model, general social support (α = 0.83) was calculated as the sum of peer support and supervisor support, while the Job Stress (i.e., job strain) indicator is calculated using the equation: JS = (Demands*2)/Job control, with values higher than 1.0 being indicative of an imbalance between perceived demands and control at work.

In the third section, the Stress in Driving Assessment Scale (EAE-C) is used to measure driving stress [44]. The scale is a 35-item Likert instrument (0–3 scale) used to assess different stressful events while driving. In its application to the Spanish-speaking population, it has been found that the instrument measures two factors: stress derived from the relationship with external factors and potential stressful driving situations (19 items, α = 0.90), and relationships with other drivers or road users in general (16 items, α = 0.82). The instrument can also yield a global coefficient of “driving stress” by means of factorial analysis, reducing the items into a single dimension.

The fourth section assesses risk predisposition using the Risky Driving Style sub-scale of the Multidimensional Driving Style Inventory (MDSI) [26]. Overall, this inventory studies driving-style factors in a non-exclusive set of eight theoretical ways to perform the driving task. The MDSI questionnaire is a Likert scale in which the participant is asked to indicate to what extent they identify with a series of situations or behaviors likely to occur when driving a vehicle (1 = Not at all, 2 = A little, 3 = Somewhat, 4 = A lot, 5 = Too much). For this paper, the risky driving style scale (five items; α = 0.83) was used to study the risk predisposition of public transport drivers through a personality-based approach.

Finally, the Driving Anger Scale (DAS) [45] was used. This is a Likert-type self-reported scale in which the participant is asked to report the intensity of anger they experienced in everyday driving situations. It is designed to assess the likelihood of experiencing anger while driving a vehicle. Items are presented on a five-level scale (1 = Not at all, 2 = A little, 3 = Somewhat, 4 = A lot, 5 = Too much). The reduced 14-item version has been previously applied in the Hispanic and English-speaking population, finding a single factor commonly known as “driving anger” (Cronbach’s Alpha coefficients between α = 0.84 and α = 0.89 in previous applications).

### 2.3. Procedure, Design, and Ethics

For this cross-sectional study, the participants were chosen according to the non-random selection of public transport drivers from different Colombian transport companies who were invited to participate. All professional drivers involved were asked to voluntarily complete questionnaires on paper during a one-hour period (approximately) provided by the companies. They were informed of their rights and the protection of their personal information by an informed consent form, emphasizing the fact that the data would only be used for research purposes. The global response rate was around 92%.

### 2.4. Statistical Analysis (Data Processing)

In addition to the descriptive analyses, a correlation analysis was performed to establish potential relationships between the study variables. The association between driving experience, hourly intensity, job strain, driving stress, risk predisposition, traffic sanctions, and the mediation of driving anger, were tested using path analysis (Structural Equation Modeling (SEM) with maximum likelihood estimations), with significance parameters: *p* < 0.05, *p* < 0.01, and *p* < 0.001. All statistical analyses were performed using SPSS (Statistical Package for Social Sciences, version 24.0 (2016) (IBM, Armonk, NY, USA), and ©IBM SPSS AMOS, version 22.0, (IBM, Armonk, NY, USA), specifically used for conducting structural analyses.

## 3. Results

### 3.1. Descriptive Results

Regarding working (driving) hours per week, or hourly intensity, an astonishing figure was found, which is worth a separate analysis: the average driving intensity of the overall sample was *M* = 72.58 (*SD* = 9.15). However, the highest mean was found for city bus drivers, with *M* = 75.39 (*SD* = 4.05) h per week; followed by taxi drivers with a similar although slightly lower mean, of *M* = 71.40 (*SD* = 10.67) h; and finally, inter-urban bus operators with a mean value of *M* = 65.34 (*SD* = 13.33) driving hours every week.

The workplace stress indicator of the JDC model (i.e., job strain) presented a mean value of *M* = 0.879 (Minimum: 0.27–Maximum: 3). In this regard, and realizing a specific frequency analysis for drivers presenting a value greater than 1.0 (ergo, presenting job strain), it was found that 20.8% of the drivers in the full sample reported this condition. In a specific analysis (by type of vehicle driven), it was found that the frequency (positive cases) for city bus drivers was 38%, 31.3% for inter-urban bus operators, and only 6.3% of taxi drivers present a job strain indicator ≥1.0.

Regarding driving stress, the mean score of the sample (scale 0–3) was *M* = 1.06 (*SD* = 0.53; Minimum: 0–Maximum: 2.90), and risk predisposition (scale 1–5) was *M* = 1.25 (*SD* = 0.52). The mean item-score obtained for driving anger (scale 1–5) was *M* = 1.85 (*SD* = 0.64; Minimum: 1–Maximum: 5). Finally, the mean for self-reported traffic sanctions received in the last two years was *M* = 1.51 (*SD* = 1.91; Minimum: 0–Maximum: 22), as shown in Table 1.

### 3.2. Bivariate Correlations

The correlation analysis (Pearson) identified many significant associations between study variables, as shown in Table 1. Specifically, driving experience was negatively and significantly related to risk predisposition, driving anger, and the self-reported rate of traffic sanctions. Hourly intensity was positively correlated to job strain and traffic sanctions, and negatively to risk predisposition and driving anger, whereas for driving stress, significant associations were found when crossing with job strain (+), risk predisposition (+), and driving anger (+). Risk predisposition was also associated with driving anger (+) and traffic sanctions (+). Finally, a significant association was found between driving anger and the rate of traffic sanctions received in the last two years (+).

### 3.3. Structural Equation Modeling

**Figure 1:**
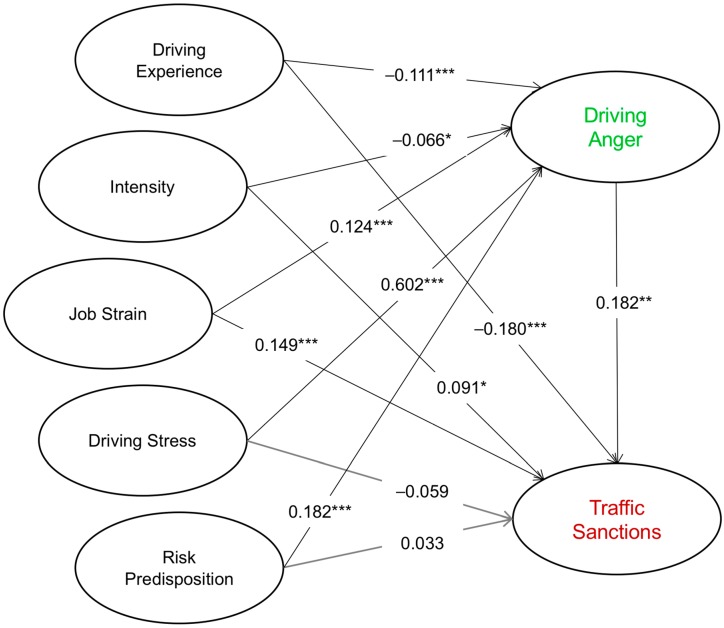
Graphic presentation of the Structural Equation Model (SEM). *** Significant at level 0.001; ** Significant at level 0.01; * Significant at level 0.05.

The structural equation model (*x*^2^(5) = 47.6234, *p* < 0.001; NFI (Normed Fit Index) = 0.910; CFI (Comparative Fit Index) = 0.915; RMSEA (Root Mean Square Error of Approximation) = 0.104) presented in Table 2, and graphically in Figure 1, shows that driving experience (β = −0.180 ***) has a direct, negative effect on the rate of traffic sanctions received in the last two years (dependent variable), while the explicative association of hourly intensity (β = 0.091 *), job strain (β = 0.149 ***), and driving anger (β = 0.182 **) occurs in a positive direction. On the other hand, neither driving stress (β = −0.059 ^n/s^) nor risk predisposition (β = 0.33 ^n/s^) had a significant explanatory effect on traffic sanctions.

As for the hypothesized mediating role of driving anger, tested in this procedure, results showed that this variable mediates the link between driving experience (β = −0.111 ***), intensity (β = −0.066 *), job strain (β = 0.124 ***), driving stress (β = 0.602 ***), risk predisposition (β = 0.182 ***), and traffic sanctions (explained variable). That is, for the case of the first three, it implies the existence of a partial mediation, and a full mediation for the last two, as shown in Table 2. In other words, driving stress and risk predisposition of drivers need driving anger to acquire a statistically significant relationship with traffic sanctions reported in a period of two years.

## 4. Discussion

The first objective of this research was to examine the association between work-related psychosocial stressors, individual characteristics of public transport drivers, and their traffic sanctions, hypothesizing that these variables have significant associations between them. In this regard, the correlational analysis showed how traffic sanctions were positively associated with the hourly intensity of driving, risk predisposition, work stress (job strain index), and driving anger. On the other hand, a negative association was found between traffic sanctions and driving experience, which, according to the evidence, may play a “protective” effect to the extent that which it strengths the automation of driving skills, contributing to the avoidance of potentially risky behaviors that may result in crashes or traffic sanctions [46].

As for the second objective, this study examined whether driving anger was a mechanism that links work stress, individual factors, and intensity of driving with traffic sanctions among professional drivers, hypothesizing that driving age exerts a mediation between them. In short, the results observed in the resulting path model are consistent with the stress/emotion/CWB model [40,41], according to which emotional expression is the primary response to environmental stressors, and an essential motivator of the negative behavioral stress-related changes. Indeed, it was found that driving anger mediates the association between driving stress of professional drivers and traffic sanctions.

It was not surprising to find fewer total mediations than partial mediations. It is known that the psycho-physiological changes associated with stress can hinder perception, risk assessment, and decision-making [47,48]. Furthermore, according to the social exchange theory of [49], workers adapt to adverse work conditions by decreasing their level of involvement with work activities, meaning that workers under work stress are more prone to errors, omissions, and counterproductive work behaviors [43]. To this extent, it can also be expected for environmental work stressors such as job strain and driving intensity to have a direct effect on penalized driving behaviors.

On the other hand, it is also theoretically consistent that driving anger involves individual antecedents such as driving experience and risk predisposition, as well as environmental antecedents such as work stressors. Risk predisposition is a personality trait compatible with the expression of positive and negative emotions (such as anger) [26,50], while driving experience is a personal resource, which can modify both the perception of stressors and the drivers’ responses to environmental demands.

The results obtained are also consistent with research on the effect of work stressors on the performance of professional drivers [22,51,52]. In sum, the available evidence suggests that work stress can, by itself, affect the psychomotor functions necessary to drive motor vehicles safely, while environmental work stressors can increase the risk of motor-vehicle operators driving dangerously as a way to express negative stress-related emotions.

In practical terms, this study may have implications for the design of interventions focusing on the well-being and management of professional drivers’ risky behaviors at the wheel. There are numerous intervention studies on the reduction of driving anger (for a summary, see [39]), and it is known that, in general, cognitive, behavioral, and relaxation interventions are effective. However, the growing knowledge on the association between work stress and professional drivers’ performance has not been broadly used for the development of occupational safety and health interventions [51,53]. This study suggests that, in the context of professional driving, interventions are needed that, in addition to improving the management of stress-related emotions, modify the structural conditions of work associated with dangerous driving (e.g., work overtime, shift work, high passenger demand, low job control, low social and organizational support, vehicles in suboptimal conditions, poor road infrastructure). In short, the present study points out the need to integrate stress and anger management when considering driving safety and any associated intervention [24].

## 5. Conclusions

This study supports the idea that traffic penalties reported by public transport drivers are preceded by work-related, personality, and other individual factors that, when combined with driving anger (also acting as a mediator), enhance negative results on traffic sanctions that, considering they are preceded by risky road behaviors, may affect overall road safety.

### Limitations of the Study

Although our sample size was large, and all statistical parameters were accurately and positively tested during the data treatment, some specific issues present in this research should be listed as potential biasing sources. Specifically, the study is limited by its cross-sectional design and by the exclusive use of self-reported data. In this sense, our research design is subject to the potential influence of common method variance, a usual methodological concern that is attributable to the measurement method, more so than to the specific measured constructs. Particularly in cross-sectional studies, where variable information is collected to obtain measures of both predictor and criterion variables from the same source, there may be detrimental effects on the interpretation of the data if there are biased results, so the theoretical support is imperative [54].

Second, and related to the specific study variables, several investigations have found that people tend to underreport their negative driving behaviors [55,56]. However, it is also known that the correlation between self-reported driving behaviors and current driving behaviors is high [57,58,59]. Furthermore, the use of highly reliable instruments diminishes the risk of self-report bias, although it does not completely eradicate them, which could happen in the particular case of the Driving Anger Scale (DAS)—reporting a relatively lower mean that that found in other applications of the same instrument. In this case, we believe that the application of the questionnaire in the work environment may increase the social desirability and acquiescence from participants.

Also, some of the significant relationships between study variables should be considered to be compared with other existing data. Apart from the significance criterion, the degree to which variables are associated is an important fact to consider when describing (for instance) a significant correlation and (even more) conducting path analyses, which require a literature-based approach. Regarding the model statistics, although the obtained fit coefficients are good overall (NFI and CFI > 0.90), one of these measures, the Root Mean Square Error of Approximation (RMSEA = 0.104) (depending on the approach, coefficients of ≤0.05, ≤0.08, and ≤0.10) may indicate a better fit between the measurement model and the structure of the data [60]), and ours (≈0.10) remains “at the limit” or “slightly high.” For solving this issue, even considering that the RMSEA is not supported and should not be pursued as the only way of determining model fit [60], we suggest using larger samples and testing complementary models to maximize the adjustment of this coefficient.

Future research could overcome the limitations of this study by using a longitudinal design with prolonged follow-up periods and objective measurements of the stressors and driving behaviors of professional drivers. Also, the study of complementary factors of the work environment (undoubtedly broader than the set of variables used in a cross-sectional design) of public transport drivers may provide additional information on work stress dynamics.

## Figures and Tables

**Table 1:** Descriptive statistics and bivariate correlations between study variables.

Study Variable		Mean	*SD*	2	3	4	5	6	7
1	Driving Experience	18.38	9.86	0.020	−0.072	−0.038	−0.142 **	−0.165 **	−0.142 **
2	Hourly intensity	72.58	9.15	-	0.082 *	−0.080	−0.181 **	−0.154 **	0.076 *
3	Job Strain	0.879	0.28		-	0.127 **	0.144 **	0.223 **	0.203 **
4	Driving Stress	1.06	0.53			-	0.225 **	0.651 **	0.081
5	Risk Predisposition	1.25	0.52				-	0.349 **	0.101 **
6	Driving Anger	1.85	0.64					-	0.193 **
7	Traffic Sanctions (last two years)	1.51	1.91						-

** Correlation is significant at the 0.01 level (two-tailed). * Correlation is significant at the 0.05 level (two-tailed).

**Table 2:** Structural equation model (SEM) to predict traffic sanctions (two years) with driving anger as the mediating variable.

Variables in the Model			Estimate ^1^	S.E. ^2^	Std. Estimate ^3^	C.R. ^4^	*p*	
Driving Anger	←	Driving Experience	−0.011	0.003	−0.111	−3.746	***	
Driving Anger	←	Hourly Intensity	−0.007	0.003	−0.066	−2.231	0.026 *	
Driving Anger	←	Job Strain	0.45	0.11	0.124	4.073	***	
Driving Anger	←	Driving Stress	0.618	0.031	0.602	19.679	***	
Driving Anger	←	Risk Predisposition	0.183	0.032	0.182	5.798	***	
Traffic Sanctions	←	Driving Experience	−0.02	0.007	−0.102	−2.861	0.004 **	
Traffic Sanctions	←	Driving Stress	−0.114	0.107	−0.059	−1.062	0.288	
Traffic Sanctions	←	Job Strain	1.024	0.253	0.149	4.048	***	
Traffic Sanctions	←	Hourly Intensity	0.019	0.007	0.091	2.571	0.01 *	
Traffic Sanctions	←	Driving Anger	0.343	0.107	0.182	3.222	0.001 **	
Traffic Sanctions	←	Risk Predisposition	0.063	0.076	0.033	0.834	0.404	

^1^ SPC = Estimated Path Coefficients (can be interpreted as linear regression weights). ^2^ S.E. = Standard Error. ^3^ Standardized Path Coefficients. ^4^ C.R. = Critical Ratio. *** Significant at level 0.001; ** Significant at level 0.01; * Significant at level 0.05.

## References

[1] Soedhoho S. (2017). Public transportation development and traffic accident prevention in Indonesia. IATSS Res..

[2] Adriazola C. Boom and Bus: How Public Transport Can Curb Road Deaths as Our Cities Grow. http://go.uv.es/MmOxf31.

[3] Gopalakrishnan S. (2012). A Public Health Perspective of Road Traffic Accidents. J. Fam. Med. Prim. Care.

[4] Boada-Grau J., Sanchez-Garcia J.C., Prizmic-Kuzmica A.J., Vigil-Colet A. (2012). Health and safety at work in the transport industry (TRANS-18): Factorial structure, reliability and validity. Span. J. Psychol..

[5] Biggs H., Dingsdag D., Stenson N. (2009). Fatigue factors affecting metropolitan bus drivers: A qualitative investigation. Work.

[6] Santos J., Lu J. (2016). Occupational Safety Conditions of Bus Drivers in Metro Manila. Int. J. Occup. Saf. Ergon..

[7] Gómez V., Cendales B., Useche S., Bocarejo J.P. (2018). Relationships of working conditions, health problems and vehicle accidents in bus rapid transit (BRT) drivers. Am. J. Ind. Med..

[8] Useche S., Alonso F., Cendales B., Autukevičiūtė R., Serge A. (2017). Burnout, Job strain and road accidents in the field of public transportation: The case of city bus drivers. J. Environ. Occup. Sci..

[9] Öz B., Özkan T., Lajunen T. (2010). Professional and non-professional drivers’ stress reactions and risky driving. Transp. Res. Part F Traffic Psychol. Behav..

[10] Tse J.L.M., Flin R., Mearns K. (2006). Bus driver well-being review: 50 Years of research. Transp. Part F Traffic Psychol. Behav..

[11] Hege A., Perko M., Johnson A., Yu C.H., Sönmez S., Apostolopoulos Y. (2015). Surveying the Impact of Work Hours and Schedules on Commercial Motor Vehicle Driver Sleep. Saf. Health Work.

[12] Useche S., Gómez V., Cendales B. (2017). Stress-related Psychosocial Factors at Work, Fatigue, and Risky Driving Behavior in Bus Rapid Transport (BRT) Drivers. Accid. Anal. Prev..

[13] Jones W., Haslam R., Haslam C. (2014). Measuring job quality: A study with bus drivers. Appl. Ergon..

[14] Pokorny M.L.I., Blom D.H.J., van Leeuwen P. (1987). Shifts, duration of work and accident risk of bus drivers. Ergonomics.

[15] Costa G. (2010). Shift Work and Health: Current Problems and Preventive Actions. Saf. Health Work.

[16] Useche S., Cendales B., Gómez V. (2017). Work stress, fatigue and Risk Behaviors at the Wheel: Data to assess the association between psychosocial work factors and risky driving on Bus Rapid Transit drivers. Data Brief.

[17] Philip P., Akerstedt T. (2006). Transport and industrial safety, how are they affected by sleepiness and sleep restriction?. Sleep Med. Rev..

[18] Gómez V. (2011). Assessment of psychosocial stressors at work: Psychometric properties of the Spanish version of the JCQ (Job Content Questionnaire) in Colombian workers. Rev. Latinoam. Psicol..

[19] De Lange A.H., Kompier M.A., Taris T.W., Geurts S.A., Beckers D.G., Houtman I.L., Bongers P.M. (2009). A hard day’s night: A longitudinal study on the relationships among job demands and job control, sleep quality and fatigue. J. Sleep Res..

[20] Useche S., Serge A., Alonso F., Esteban C. (2017). Alcohol Consumption, Smoking, Job Stress and Road Safety in Professional Drivers. J. Addict. Res. Ther..

[21] Habibi E., Poorabdian S., Shakerian M. (2015). Job strain (demands and control model) as a predictor of cardiovascular risk factors among petrochemical personnel. J. Educ. Health Promot..

[22] Kontogiannis T. (2006). Patterns of driver stress and coping strategies in a Greek sample and their relationship to aberrant behaviors and traffic accidents. Accid. Anal. Prev..

[23] Matthews G., Dorn L., Hoyes T.W., Davies D.R., Glendon A.I., Taylor R.G. (1998). Driver stress and performance on a driving simulator. Hum. Factors.

[24] Ge Y., Qu W., Jiang C., Du F., Sun X., Zhang K. (2014). The effect of stress and personality on dangerous driving behavior among Chinese drivers. Accid. Anal. Prev..

[25] Slavinskienė J., Žardeckaitė-Matulaitienė K., Endriulaitienė A., Markšaitytė R., Šeibokaitė L. (2016). Personality Profiles of Traffic Offenders: Does It Correlate to Alcohol Consumption?. Eur. Proc. Soc. Behav. Sci..

[26] Taubman-Ben-Ari O., Mikulincer M., Gillath O. (2004). The multidimensional driving style inventory (MDSI). Scale construct and validation. Accid. Anal. Prev..

[27] Beirness D.J. (1993). Do we really drive as we live? The role of personality factors in road crashes. Alcohol Drugs Driv..

[28] McLinton S.S., Dollard M.F. (2010). Work stress and driving anger in Japan. Accid. Anal. Prev..

[29] Deffenbacher J.L., Filetti L.B., Lynch R.S., Dahlen E.R., Oetting E.R. (2002). Cognitive-behavioral treatment of high anger drivers. Behav. Res. Ther..

[30] Mann R.E., Zhao J., Stoduto G., Adlaf E.M., Smart R.G., Donovan J.E. (2007). Road rage and collision involvement. Am. J. Health Behav..

[31] Sagar R., Mehta M., Chugh G. (2013). Road rage: An exploratory study on aggressive driving experience on Indian roads. Int. J. Soc. Psychiatry.

[32] Malta L.S., Blanchard E.B., Freidenberg B.M. (2005). Psychiatric and behavioral problems in aggressive drivers. Behav. Res. Ther..

[33] Sümer N. (2003). Personality and behavioral predictors of traffic accidents: Testing a contextual mediated model. Accid. Anal. Prev..

[34] Shinar D. (1998). Aggressive driving: The contribution of the drivers and the situation. Transp. Res. Part F Traffic Psychol. Behav..

[35] Kaiser S., Furian G., Schlemback C. (2016). Aggressive Behaviour in Road Traffic-Findings from Austria. Transp. Res. Proc..

[36] Wickens C.M., Mann R.E., Ialomiteanu A.R., Stoduto G. (2016). Do driver anger and aggression contribute to the odds of a crash? A population-level analysis. Transp. Res. Part F Traffic Psychol. Behav..

[37] Zhang T., Chan A.H.S., Zhang W. (2015). Dimensions of driving anger and their relationships with aberrant driving. Accid. Anal. Prev..

[38] Galovski T.E., Blanchard E.B. (2002). The effectiveness of a brief psychological intervention on court-referred and self-referred aggressive drivers. Behav. Res. Ther..

[39] Deffenbacher J.L. (2016). A review of interventions for the reduction of driving anger. Transp. Res. Part F Traffic Psychol. Behav..

[40] Spector P.E., Cooper C.L. (1998). A control theory of the job stress process. Theories of Organizational Stress.

[41] Spector P.E., Fox S. (2002). An emotion-centered model of voluntary work behavior: Some parallels between counterproductive work behavior and organizational citizenship behavior. Hum. Resour. Manag. Rev..

[42] Lazarus R.S. (1991). Emotion and Adaptation.

[43] Fox S., Spector P.E., Miles D. (2001). Counterproductive work behavior (CWB) in response to job stressors and organizational justice: Some mediator and moderator tests for autonomy and emotions. J. Vocat. Behav..

[44] Fernández-Seara J., Mielgo N. (1992). Escalas de Apreciación del Estrés (Stress Appreciation Scales).

[45] Deffenbacher J.L., Oetting E., Lynch R.S. (1994). Development of a driving anger scale. Psychol. Rep..

[46] Vlakveld W.P. (2005). Young, Novice Motorists, Their Crash Rates, and Measures to Reduce Them: A Literature Study.

[47] McEwen B.S., Sapolsky R.M. (1995). Stress and cognitive function. Curr. Opin. Neurobiol..

[48] Staal M.A. (2004). Stress, Cognition, and Human Performance: A Literature Review and Conceptual Framework. https://ntrs.nasa.gov/archive/nasa/casi.ntrs.nasa.gov/20060017835.pdf.

[49] Cropanzano R., Rupp D.E., Mohler C.J., Schminke M., Ferris J. (2001). Three roads to organizational justice. Research in Personnel and Human Resources Management.

[50] Lerner J.S., Keltner D. (2001). Fear, anger, and risk. J. Personal. Soc. Psychol..

[51] Taylor A., Dorn L. (2006). Stress, fatigue, health, and risk of road traffic accidents among professional drivers: The contribution of physical inactivity. Annu. Rev. Public Health.

[52] Westerman S.J., Haigney D. (2000). Individual differences in driver stress, error and violation. Personal. Individ. Differ..

[53] Aust B., Peter R., Siegrist J. (1997). Stress management in bus drivers: A pilot study based on the model of effort-reward imbalance. Int. J. Stress Manag..

[54] Podsakoff P.M., MacKenzie S.B., Lee J.Y., Podsakoff N.P. (2003). Common method biases in behavioral research: A critical review of the literature and recommended remedies. J. Appl. Psychol..

[55] Lajunen T., Summala H. (2003). Can we trust self-reports of driving? Effects of impression management on driver behaviour questionnaire responses. Transp. Res. Part F Traffic Psychol. Behav..

[56] Grayson G.B. (1997). Behavioural Research in Road Safety VII. Proceedings of a Seminar.

[57] Parker D. The relationship between speeding attitudes and speeding behaviour. Proceedings of the Seminar on Behavioural Research in Road Safety VII.

[58] Walton D. (1999). Examining the self-enhancement bias: professional truck drivers’ perceptions of speed, safety, skill and consideration. Transp. Res. Part F Traffic Psychol. Behav..

[59] West R.J. Individuals in accident risk: A review of findings and an examination of methods. Proceedings of the Behavioural Research in Road Safety VI.

[60] Chen F., Curran P.J., Bollen K.A., Kirby J., Paxton P. (2008). An Empirical Evaluation of the Use of Fixed Cutoff Points in RMSEA Test Statistic in Structural Equation Models. Sociol. Methods Res..

